# Anti-apoptotic and neuroprotective effects of Tetramethylpyrazine following spinal cord ischemia in rabbits

**DOI:** 10.1186/1471-2202-7-48

**Published:** 2006-06-14

**Authors:** Li-Hong Fan, Kun-Zheng Wang, Bin Cheng, Chun-Sheng Wang, Xiao-Qian Dang

**Affiliations:** 1 Department of Orthopedics, Second Affiliated Hospital Xi'an Jiao Tong University, Xiwu Road, Xi'an, shaanxi, 710004, China

## Abstract

**Background:**

Tetramethylpyrazine (TMP) is one of the most important active ingredients of a Chinese herb Ligusticum wallichii Franchat, which is widely used in many ischemia disorders treatments. However, the exact mechanism by which TMP protects the spinal cord ischemia/reperfusion (I/R) injury is still unknown. For this purpose, rabbits were randomly divided into sham group, control group and TMP group. After the evaluation of neurologic function, the spinal cords were immediately removed for biochemical and histopathological analysis. Apoptosis was measured quantitatively by the terminal transferase UTP nick end-labeling (TUNEL) method and confirmed by electron microscopic examination, the expression of Bax and Bcl-2 was immunohistochemically evaluated and quantified by Western blot analysis.

**Results:**

Neurologic outcomes in the TMP-group were significantly better than those in the control group (P < 0.05). TMP decreased spinal cord malondialdehyde (MDA) levels and ameliorated the down regulation of spinal cord superoxide dismutase (SOD) activity. TMP significantly reduced the loss of motoneurons and TUNEL-positive rate. Greater Bcl-2 and attenuated Bax expression was found in the TMP treating rabbits.

**Conclusion:**

These findings suggest that TMP has protective effects against spinal cord I/R injury by reducing apoptosis through regulating Bcl-2 and Bax expression.

## Background

Spinal cord ischemia/reperfusion (I/R) injury may present immediate or delayed paraplegia that occurs 4% to 33% of patients undergoing surgery on the thoracic aorta [1]. Therefore, In attempt to prevent this complication, various methods of spinal cord protection have been suggested, including temporary shunts or partial bypass, hypothermia, drainage of cerebrospinal fluid, and pharmacologic measures [2-4]. Despite their use, paraplegia remains a persistent complication[5].

Although the exact mechanism of I/R injury is not fully understood, it is believed that Oxidative stress plays a pivotal role in triggering lipid peroxidation, DNA damage and specific gene expression [6]. In addition, blood-brain-barrier disruption, mediated by oxygen free radicals, results in spinal cord edema[7]. Oxidative stress resulting from reactive oxygen species (ROS) production is also implicated in apoptosis. Although ischemic neuronal cell death had been traditionally interpreted by necrotic mechanisms, the role of apoptotic mechanisms has been recently proposed in neuronal cell death following spinal cord I/R injury [8]. Several studies have suggested that apoptotic mechanisms were initiated at the molecular level in I/R neural cells[9,10].

In traditional Oriental medicine, Ligusticum wallichii Franchat (Chuan Xiong) is applied in the treatment of neurovascular and cardiovascular diseases. Tetramethylpyrazine (TMP), a purified and chemically identified component of Chuan Xiong, has strong effects to scavenge oxygen free radicals [11]. It has been shown that TMP can alleviate kidney and brain damage induced by I/R via scavenging free radicals[12,13]. However it remains uncertain whether the protective effects of TMP on spinal cord I/R injury are related to scavenging free radicals and suppressing apoptotic pathways.

In this study, the authors investigated the effect of TMP on the neurologic function, biochemical and histopathological changes and studied its impact on expression of pro- and anti-apoptotic proteins as well as the numbers of apoptotic cells following spinal cord I/R injury in rabbits.

## Methods

All experimental protocols were approved by our Institutional Committee on Animal Research, and were carried out in accordance with the National Institutes of Health guidelines for animal use and care (National Institutes of Health publication no. 96- 23, revised 1996). Experiments were performed on 36 adult male New Zealand White rabbits (provided by Experimental Animal Center of the Xi'an Jiaotong University) weighing 2.5 to 3.0 kg. The animals were initially anaesthetised with pentobarbital sodium (30 mg/kg IV, sigma, USA, NO: 20030709), followed by a half-dose as required during surgical procedure. No animals received hemodynamic or ventilatory support. The left ear vein was cannulated with a 24-gauge catheter for intravenous drug administration. The right femoral artery was catheterized for blood pressure and heart rate monitoring (Spacelab, USA, model 90206A). Arterial blood was sampled for determination of blood gases (AVL-2, Switzerland) and blood glucose (One Touch II, USA). The rectal body temperature was maintained close to 38°C with the aid of a heating pad during the study.

### Experimental groups and Animal models

Rabbits were randomly assigned to 3 groups (n = 12 each). In the TMP group, TMP (30 mg/kg) (Changzhou Pharmacological Co., China, NO: 99091401) was injected via ear vein 30 min before aortic clamping and at the onset of reperfusion. Control animals underwent standard aortic occlusion and intravenous injection of 0.9% sodium chloride under conditions identical to the TMP injection. Sham operated animals subjected to operative dissections without aortic occlusion.

Each group of animals was divided into four experimental subgroups: group A for Biochemical analysis (n = 3), group B for hematoxylin and eosin staining (H&E), Terminal Deoxynucleotidyltransferase-Mediated dUTP Nick End-Labeling (TUNEL) staining and immunohistochemistry (n = 3), group C for electron microscopy (n = 2), group D for Western blot assay (n = 4). The rabbit model of spinal cord I/R injury was established according to Savas'discription [14]. Briefly, after sterile preparation, a 10-cm midline incision was performed. Following anticoagulation with 400 unit's heparin, the abdominal aorta was cross-clamped at the level just inferior to the origin of the left renal artery and at the level of aortic bifurcation for 30 min. Reperfusion was initiated by removal of the occlusion and lasted 48 h. The abdomen was then closed.

### Neurologic evaluation

Neurological function was observed at the 24th and 48th hour after reperfusion according to Johnson's score[15].

0: Hind-limb paralysis;

1: Severe paraparesis;

2: Functionalmovement, no hop;

3: Ataxia, disconjugate hop;

4: Minimal ataxia;

5: Normal function.

Two individuals without knowledge of the treatment graded neurological function independently.

### Histological study

The animals were euthanized by intravenous administration of a high concentration of pentobarbital at the 48th hour and the spinal cords were quickly removed. The spinal cords were immersed in 4% paraformaldehyde in 0.1 mol/l phosphate buffer and stored at 4°C for 2 weeks. The specimens for microscopy were prepared by obtaining spinal cord cross sections from the L2 or L3 vertebra. The specimens were then embedded in paraffin, cut into sections of 5μm thickness, stained with hematoxylin-eosin (H&E). The specimens were examined under the light microscope by a neuropathologist who was blinded to the study.

### Preparation for electron microscopic examination of excised cords

The specimens were fixed in 2.5% glutaraldehyde for 6 h, washed in phosphate buffer (pH 7.4), postfixed in 1% osmium tetroxide in phosphate buffer (pH 7.4), and dehydrated in increasing concentrations of alcohol. Then the tissues were immersed in propylene oxide and embedded in epoxy resin embedding media. Ultrathin sections (thickness 60 nm) were cut and stained with uranyl acetate and lead citrate, and examined with a ZEISS-EM902 transmission electron microscope (Carl Zeiss, Thornwood, NY).

### Biochemical analysis

Spinal cord tissues were washed two times with cold saline solution and stored in a deep freeze kept at -30°C until analysis. Tissue MDA levels were determined by the method described by Wasowicz[16]. Briefly, MDA was reacted with thiobarbituric acid by incubating for 1 h at 95–100°C. Following the reaction, fluorescence intensity was measured in the n-butanol phase with a fluorescence spectrophotometry(Hitachi, Model F-4010, Japan), by comparing with a standard solution of 1,1,3,3 tetramethoxypropane. Results were expressed in terms of nmol/g wet tissue. Total (Cu-Zn and Mn) SOD activity was measured by reduction of nitrobluetetrazolium (NBT) by xanthine-xanthine oxidase system. Enzyme activity leading to 50% inhibition was accepted as one unit. Results were expressed as U/mg protein [17]. Protein concentrations were determined according to Lowry's method [18].

### TUNEL staining and immunohistochemistry

TUNEL staining was performed on paraffin sections using an in situ cell death detection kit (Rochev, Germany) according to the manufacturer's instructions. Sections were counterstained with hematoxylin. A negative control was similarly performed except for omitting TUNEL reaction mixture. Only cells showing nuclear condensation/fragmentation and apoptotic bodies in the absence of cytoplasmic TUNEL reactivity were considered apoptotic. For immunohistochemistry, sections, blocked using 2% normal goat serum in PBS, were incubated for overnight at 4°C with mouse monoclonal antibody against Bcl-2/Bax at a dilution of 1:50 (Maxim Biotech Inc, China) followed by followed by a biotinylated sheep anti-mouse antibody and avidin-biotin complex (Vector Laboratories, Burlingame, CA, USA.) for 2 h. The slices were colorized with DAB/H_2_O_2 _solution, and then cell nucleuses were counterstained with hematoxylin. Each procedure was followed by several rinses in PBS. Blank staining was carried out in the same way as the above, except for eliminating the primary antibodies. Brown color of nuclei was taken as the positive staining of apoptotic neuronal cells and Brown color of cytoplasm was taken as the positive staining of Bcl-2/Bax. For quantitative analysis, 10 microscopic fields were taken, and all neurons, including neurons with TUNEL staining were counted. The mean values of the percentage of neurons with TUNEL positive staining were taken for further processing.

### Western blot assay of Bcl-2 and Bax proteins

Spinal cord tissue was placed in lysis buffer containing inhibitors(leupeptin, pepstatin A, and aprotinin), homogenized, and then centrifuged(12,000 × g). After determining concentration of protein in each sample using a protein assay (Bio-Rad, Hercules, CA, USA), Samples were loaded (50 mg of protein/lane), electrophoresed on a 15% SDS-polyacrylamide gel electrophoresis (SDS-PAGE) gel and blotted to a nylon filter. Blots were probed with mouse monoclonal antibody against Bcl-2/Bax at a dilution of 1:200 (Maxim Biotech Inc, China) and visualized with horseradish peroxidase-conjugated secondary antibodies by enhanced chemiluminescence detection reagents (Amersham). Bcl-2 and Bax proteins were detected as 26 and 21 kDa bands, respectively, using molecular weight marker bands. The filter was scanned by FluorImager 595 (Amersham) and quantified with NIH Image J.

### Statistical analysis

Statistical analysis was performed using SPSS 10.0. An unpaired t-test was used for comparisons in physiological parameters, MDA levels, SOD activity, TUNEL-positive rate and Bcl-2/Bax expression between the groups. Neurological scores were analyzed with nonparametric method (Kruskal-Wallis test) followed by the Mann-Whitney U test with Bonferroni correction. Data were expressed as mean ± S.D. and statistical significance was set at *P *<0.05.

## Results

### Physiological parameters

Physiological variables were within normal limits at any evaluating time points, and showed no statistically significant differences between the groups [see Additional file 1].

### Neurologic function evaluation

The results are shown in Table 1. No neurologic anomaly was observed at the 24th and 48th hours after reperfusion in sham group, except a mild alteration in one animal. The values of Johnson's score in TMP group and control group were significantly lower in comparison with sham group at the 24th hour. The values of control group were significantly lower at the 48th hour in comparison with the same group. Another finding was that, at both the 24th and 48th hour, the values of the TMP group were significantly better in comparison with the control group.

### Histopathologic study

**Figure 1:**
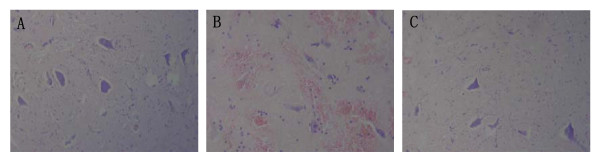
**Spinal cord histopathology following I/R**. Rabbits were treated with vehicle (saline) or TMP (30 mg/kg) prior to the onset of ischemia (30 min), followed by 48 h of reperfusion. Sham animals received the same pretreatment as I/R group, followed by sham operation. The ischemic spinal cord sections were prepared and stained with H&E. Figure A represent the sham rabbits, which show normal histology. Figure B represent control rabbits, which show a pattern of necrotic changes with karyolysis and neurophil vacuolation. Figure C represent TMP- treated rabbits, which show mild degrees of destruction such as triangular shape, and Nissl granule loss in some motor neurons. Figures(magnification × 200) are representative of 3 separate experiments with similar results.

Representative photographs of HE-stained sections are shown in Fig 1. No sign of histopathologic abnormalities was observed in sham-operated rabbits with normal motor function (Figure1A). However, the spinal cords from rabbits in control group that suffered paraplegia (Johnson score 1) exhibited necrotic changes with karyolysis and neurophil vacuolation (Figure1B). The spinal cords of the rabbits rated Johnson score 4 in TMP group showed mild degrees of destruction such as triangular shape, and Nissl granule loss in some motor neurons (Figure1C).

### Electron microscopy

**Figure 2:**
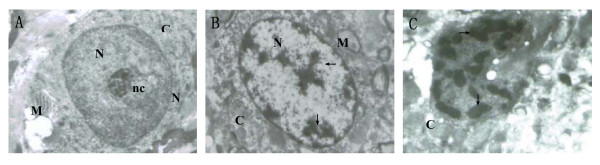
**Transmission Electron microscopic evidence of neuronal apoptosis in the ventral horn of the spinal cord**. The rabbit spinal cords were fixed by transcardial perfusion and removed at 48 h reperfusion, or operation for sham control and processed as described in Experimental Procedures. A, sham control (magnification × 10000); B-C, I/R control (magnification × 20000). I/R induced neuronal apoptosis, as demonstrated by specific morphological features. No apoptotic neurons were found in sham group sections. N, nucleus; nc, nucleolus; C, cytoplasm; M, mitochondria. Figures are representative of 3 separate experiments with similar results.

Under transmission electron microscopy, nucleus of neuron in sham group displayed normal morphology, including normal shape of nuclei and evenly distributed nuclear chromtin(Figure2A). In control group, neuron showed features of apoptosis, including nucleus shrinkage, dense aggregation of chromatin (Figure2B, arrows) and chromatin margination (Figure2C, arrows),. The results in Figure2B and Figure2C indicate that neural cell apoptosis occurred in the spinal cord at 48 h following 30 min ischemia.

### The biochemical analysis of oxidant stress markers in spinal cord

**Figure 3:**
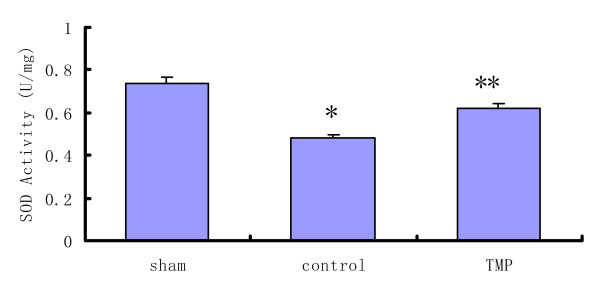
**Effects of TMP on SOD Activity at the 48th hour reperfusion**. The Cu/Zn-SOD activity of spinal cord in sham group, Control group and TMP group was determined as described under "Methods". Average value and SD are shown, N = 3.*P < 0.01, vs sham group; **P < 0.01, vs control group.

**Figure 4:**
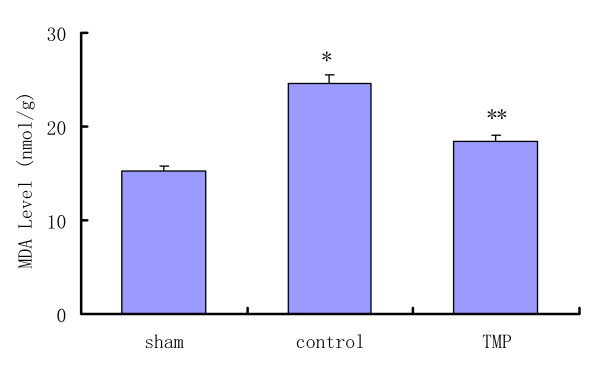
**Effects of TMP on MDA Level at the 48th hour reperfusion**. The MDA level of spinal cord in sham group, Control group and TMP group was determined as described under Methods. Average value and SD are shown, N = 3.*P < 0.01, vs sham group; **P < 0.01, vs control group.

A significant decrease in SOD activities in the control group was determined when compared to that of sham group (p < 0.01). TMP treatment significantly prevented the decreases in the SOD activities produced by I/R (Figure3). I/R produced a significant increase in MDA level in spinal cord when compared with sham group (p < 0.01). I/R-induced increments in MDA content were significantly prevented by TMP Treatment(Figure 4).

### TUNEL staining and immunohistochemistry for Bax and Bcl-2

**Figure 5:**
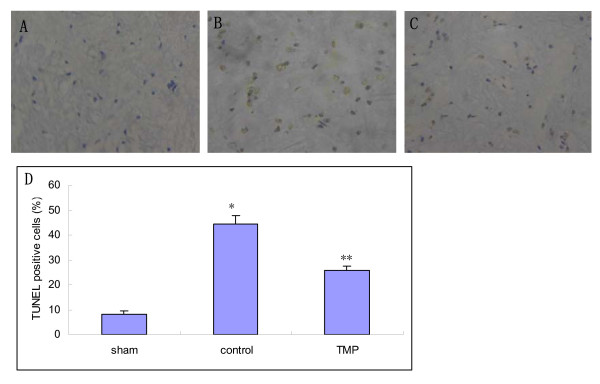
**Effects of TMP on cell apoptosis in the spinal cord at the 48th hour reperfusion**. Representative images of TUNEL staining (magnification × 200) in sham group (A), Control group (B) and TMP groups (C). Quantitative analysis of apoptosis rate (D). Cell apoptosis was determined using TUNEL staining as described under "Methods". Cell apoptosis rate is expressed as the mean ± S.D. from three experiments. *P < 0.01, vs sham group; **P < 0.01, vs control group.

**Figure 6:**
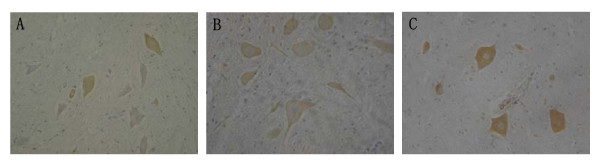
**Effects of TMP on Bax expression in spinal cord at the 48th hour reperfusion**. Immunohistochemical photomicrographs (magnification × 400) of anterior horn tissue stained for Bax protein in sham group (A), control group (B) and TMP group (C). Immunostaining was performed using a specific anti- Bax antibody and developed with stable DAB. The positive staining of Bax is presented by a brown color of cytoplasm. Figures are representative of 3 separate experiments with similar results.

**Figure 7:**
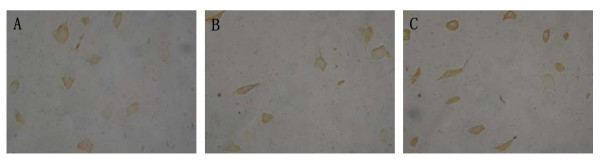
**Effects of TMP on Bcl-2 expression in spinal cord at the 48th hour reperfusion**. Immunohistochemical photomicrographs (magnification × 400) of anterior horn tissue stained for Bcl-2 protein in sham group (A), control group (B) and TMP group (C). Immunostaining was performed using a specific anti- Bcl-2 antibody and developed with stable DAB. The positive staining of Bcl-2 is presented by a brown color of cytoplasm. Figures are representative of 3 separate experiments with similar results.

No TUNEL-positive cells were detected in sham group (Figure5A), whereas many cells were intensely stained in the anterior horn of spinal cord after I/R (control group) (Figure5B). However, TMP treatment decreased staining and reduced the number of TUNEL-positive cells (Figure5C). Representative microphotograph of immunohistochemistry staining for Bax and Bcl-2 are shown in Figure 6, 7 respectively. The expression of Bax was weak in the sham group (Figure6A) and more Bax-positive neurons in the control group (Figure6B) than in TMP-treated animals (Figure6C). The expression of Bcl-2 was strong in sham group (Figure7A) and moderate Bcl-2 expression in the control group (Figure7B) compared with the strong up-regulation of Bcl-2 in the TMP-treated group (Figure7C).

### Expression in Bcl-2 and Bax proteins

**Figure 8:**
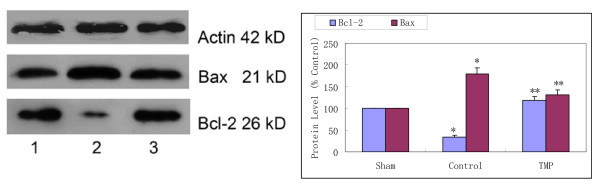
**Effect of TMP on expression of Bcl-2/Bax proteins in spinal cord at the 48th hour reperfusion**. Western analysis was carried out as described under Methods and the blots are shown in the upper right hand corner. Lane 1 represents sham group; Lane 2 represents vehicle control group; Lane 3 represents TMP group. The bars depict densitometry analyses of Western Blots from four independent experiments. Ischemia reperfusion obviously reduced Bcl-2 expression and increased Bax expression compared with the sham group. *P < 0.01, vs sham group; **P < 0.01, vs control group.

Expression of Bcl-2 and Bax proteins was visualized by Western blot analysis as shown in Figure 8. Spinal cord ischemia reperfusion obviously reduced Bcl-2 expression and increased Bax expression compared with the sham group. Treatment with TMP was associated with greater Bcl-2 and attenuated Bax expression relative to the vehicle control group.

## Discussion

### Neuroprotective effects of TMP

This study demonstrates a considerable neuropotective effect of TMP, an active ingredient of the Chinese herb Ligusticum wallichii Franchat, on neurological, biochemical and histopathological status of spinal cord I/R in rabbits. There is increasing evidence that free radicals are generated by I/R and they contribute to tissue injury [19]. ROS attack a variety of critical biological molecules, including membrane lipids, essential cellular proteins, and DNA[20]. We studied the effect of TMP on lipid peroxidation, which was measured in terms of MDA. TMP reversed the increase in MDA levels to a considerable extent, thereby confirming its antioxidant role in I/R. Furthermore, we showed that SOD levels increased following TMP treatment. The SOD is the first line of defense against free radical generation. It has been reported that total SOD is down-regulated following spinal cord I/R [21]. Decreased SOD renders a tissue susceptible to oxidant injury. Therefore, the elevated SOD levels induced by TMP may contribute to reduce superoxide radicals following spinal cord I/R.

In our study, the histology of the spinal cords confirms the clinical observations. In general, severity of injury correlated well with the degree of neuronal damage. In animals that had significant impairment of motor function, evidence of both necrosis and apoptosis was apparent. However, TMP increased the proportion of animals that had normal motor function, and in these animals, necrosis was decreased and more normal motoneurons were preserved. This improvement of neurologic function and the histopathological findings reveal the protective effect of TMP on spinal tissue against I/R injury.

### Bax/Bcl-2 dependent anti-apoptotic effects of TMP

The principal finding of this work is that TMP increased Bcl-2 expression together with significant decrease in Bax expression in spinal cord. In addition, TMP significantly reduced the number of TUNEL-positive cells in anterior horn of the spinal cord, and the Bax/Bcl-2 expression appeared to correlate with the anti-apoptotic effect.

It has been suggested that neuronal apoptosis occurs concurrently with necrosis following spinal cord I/R and may contribute predominantly to delayed onset of neuronal cell death [22,23]. The major mechanism of I/R induced apoptosis is attributed to the ROS release. ROS induces apoptosis by causing DNA damage, oxidation of lipid membranes, and activation of the proteins responsible for apoptosis[24,25]. Among these apoptosis regulatory proteins, the Bcl-2 family consists of both cell death promoters and cell death preventers. The ratio of anti- to pro-apoptotic molecules such as Bcl-2/Bax determines the response to a death signal. Indeed, the role of the Bcl-2 family in regulating apoptosis has been characterized in CNS ischemia[26,27]. In addition, over-expression of Bcl-2 may play a protective role in neuropathological sequelae after CNS insults [28].

Recent studies have revealed that antioxidants attenuated ischemic neuronal apoptosis through Bcl-2 up-regulation parallel to Bax down-regulation [29]. TMP has been reported to attenuate oxidative damage and apoptosis both in vitro and in vivo [30,31]. In the present study, treatment with TMP is related to an up-regulated level of the anti-apoptotic protein Bcl-2 and a down-regulated pro-apoptotic protein Bax, suggesting that TMP exhibit an inhibitory effect on apoptotic cell death due to spinal cord I/R through modulation of Bcl-2 family.

## Conclusion

TMP shows a potent protection against spinal cord I/R injury in rabbit model, and reduces apoptotic cell death through Bcl-2 up-regulation parallel to Bax down-regulation.

## Authors' contributions

Li-Hong Fan carried out all in vivo studies, participated in the design of the study and contributed to manuscript preparation. Kun-Zheng Wang assisted in the design of the study, reviewed all data, and assisted in writing the manuscript. Bin Cheng assisted in histopathologic analysis and neurological testing. Chun-Sheng Wang performed all the statistical analysis. Xiao-Qian Dang helped the design of the study and participated in writing the manuscript. All authors have read and approved the final manuscript.

## Supplementary Material

Additional File 1 Pysiologic Variables MABP: mean arterial blood pressure; RT: rectal temperature; BG: blood glucose level. There were no statistically significant differences in any physiological parameters between the groups. Click here for file

## Figures and Tables

**Table 1:** Changes in neurologic outcome at the 24 th and 48th hour reperfusion

Animal groups	n	Motor score						Average motor score
			
		0	1	2	3	4	5	
Sham group								
24 h	12						12	5
48 h	12					1	11	4.92 ± 0.29
Control group								
24 h	12			6	4	2		2.67 ± 0.78 *
48 h	12		5	5	2			1.75 ± 0.75 *,***
TMP group								
24 h	12		2	3	7			3.42 ± 0.79*, **
48 h	12		2	5	5			3.25 ± 0.75*, **

*P < 0.01 when compared with sham group; **P < 0.05 when compared with control group. *** P < 0.05 when compared with the same group at the 24 th hour.
